# Beyond Smoking: Exploring Etiotypes of Chronic Obstructive Pulmonary Disease (COPD) Using the Global Initiative for Chronic Obstructive Lung Disease (GOLD) 2023 Classification

**DOI:** 10.7759/cureus.94579

**Published:** 2025-10-14

**Authors:** Ananthu Sobhanan, Shrinath V, Samruddhi Deshpande, Rahul Tyagi, Vikas Marwah

**Affiliations:** 1 Respiratory Medicine, Army Institute of Cardiothoracic Sciences, Pune, IND; 2 Respiratory Medicine, INHS Asvini, Mumbai, IND

**Keywords:** air pollution, asthma copd overlap, biomass smoke smoking-related copd, chronic obstructive pulmonary disease, copd, etiotypes of copd, gold 2023 classification, gold copd, india, non-smoking copd

## Abstract

Background and objective

Chronic obstructive pulmonary disease (COPD) is increasingly recognized as a heterogeneous condition with multiple etiologies beyond smoking. The Global Initiative for Chronic Obstructive Lung Disease (GOLD) 2023 guidelines define seven distinct COPD etiotypes based on underlying risk factors. This study aimed to identify the distribution of these GOLD-defined COPD etiotypes in an Indian tertiary care cohort and examine their association with disease severity, symptom burden, and exacerbation frequency.

Methods and results

In this cross-sectional record-based study, 1,452 medical records (Jan 2020-May 2023) were screened, identifying 86 patients with spirometry-confirmed COPD. Etiotypes were assigned per GOLD 2023 definitions. Among these patients, 61.6% had multiple etiotypes. Male patients predominantly exhibited smoking- and pollution-related COPD, while all female patients had pollution-related COPD. A significant association was found between multiple etiotypes and greater spirometric severity (p = 0.046), though no significant links were observed with symptom burden or exacerbation frequency.

Conclusion

COPD in the Indian population demonstrates diverse and often overlapping etiological profiles, with non-smoking exposures such as biomass and incense smoke playing a major role, particularly in women. The presence of multiple etiotypes correlates with worse lung function, underscoring the importance of population-specific phenotyping and routine spirometry for optimal COPD management.

## Introduction

Chronic obstructive pulmonary disease (COPD) is a progressive and heterogeneous respiratory condition characterized by chronic symptoms and persistent airflow limitation resulting from structural abnormalities in the airways and alveoli. Its pathogenesis involves sustained inflammation, oxidative stress, and protease-antiprotease imbalance within the lungs [1].

Tobacco smoking is a well-known risk factor for COPD, contributing to over 50% of the global population-attributable risk [2]. However, recent data indicate that 25-45% of COPD cases occur in never-smokers, particularly in low- and middle-income countries. These cases are often linked to environmental and occupational exposures, such as household biomass smoke, outdoor air pollution, industrial dust, poorly controlled asthma, pulmonary infections, impaired lung development, and low socioeconomic status [3]. India, with its high burden of indoor pollution and lower sociodemographic index (SDI), reports a disproportionately high prevalence of non-smoking-related COPD [2]. Although never-smokers typically present with milder spirometric impairment, they remain susceptible to disease progression and exacerbations. Genetic predispositions, such as alpha-1 antitrypsin deficiency, further contribute to COPD pathophysiology in specific subgroups [4].

Recognizing the limitations of a one-size-fits-all approach, the Global Initiative for Chronic Obstructive Lung Disease (GOLD) 2023 guidelines introduced a classification framework comprising seven distinct etiotypes: COPD-G: Genetically determined (e.g., alpha-1 antitrypsin deficiency), COPD-D: Developmental (e.g., prematurity, low birth weight), COPD-C: Cigarette or cannabis smoke exposure, COPD-P: Indoor/outdoor air pollution or biomass smoke exposure, COPD-I: Infection-related (e.g., tuberculosis, childhood respiratory infections, HIV), COPD-A: Asthma-COPD overlap, COPD-U: Unknown cause [5]. Importantly, these etiotypes are not mutually exclusive and may co-exist in a single patient, influencing disease severity and progression.

The treatable traits concept, which emphasizes personalized care based on clinical phenotypes, has further highlighted the need for refined classification [6]. To date, no studies have systematically evaluated the distribution of GOLD-defined etiotypes within the Indian population or explored their associations with clinical outcomes such as lung function, symptom burden, or exacerbation frequency. This study aims to address this gap by classifying COPD patients based on GOLD 2023 etiotypes and analyzing their relationship with spirometric severity and symptom profiles in an Indian tertiary care setting.

## Materials and methods

Study design and objectives

This was a cross-sectional, retrospective observational study conducted at a tertiary care center in India. Electronic medical records from January 2020 to May 2023 were consecutively screened to identify patients diagnosed with COPD. The primary objective was to classify patients according to the etiotype framework defined in the 2023 GOLD guidelines and to examine associations between etiotypes and spirometric severity, breathlessness, and exacerbation frequency.

Inclusion and exclusion criteria

Patients were eligible if they had a diagnosis of COPD confirmed by a pulmonologist based on clinical and/or radiological features, along with spirometric evidence of persistent post-bronchodilator airflow obstruction which was confirmed according to post-bronchodilator forced expiratory volume in 1 s (FEV₁)/forced vital capacity (FVC) < 0.70 as per GOLD 2023. This ensures diagnostic standardization.. Exclusion criteria included incomplete medical records, inability to perform spirometry, or refusal to provide informed consent. The study was approved by the Institutional Ethics Committee of the Armed Forces Medical College, Pune (IEC/2023/420). Written informed consent was obtained from all participants.

Data collection and etiotype classification

Patient records were identified using keyword searches related to COPD. Records with spirometry-confirmed airflow obstruction and consistent clinical or radiological findings were included. Eligible patients were contacted for a detailed history of environmental and occupational exposures to aid etiotype classification. Etiotypes were defined as per GOLD 2023 guidelines. Patients were categorized as having either a single or multiple etiotypes; therefore, classification was additive rather than hierarchical, permitting assignment to multiple etiotypes in cases of overlapping exposures.

Clinical assessments and statistical analysis

Spirometric severity was classified according to GOLD staging: GOLD 1: FEV₁ ≥ 80%, GOLD 2: FEV₁ 50-79%, GOLD 3: FEV₁ 30-49%, GOLD 4: FEV₁ < 30%. Breathlessness was assessed using the modified Medical Research Council (mMRC) dyspnea scale: mild to moderate: grades 1-2 and severe to very severe: grades 3-4. Exacerbation history was categorized as frequent: two or more exacerbations in the previous year, and non-frequent: fewer than two exacerbations. Data were analyzed using Microsoft Excel (Microsoft, Redmond, WA, USA) and SPSS version 23.0 (IBM Corp., Armonk, NY, USA). Continuous variables were reported as means ± standard deviation (SD); categorical variables were expressed as frequencies and percentages. Chi-square tests were used to evaluate associations between etiotype classification and clinical severity indicators. A p-value <0.05 was considered statistically significant.

## Results

**Figure 1:**
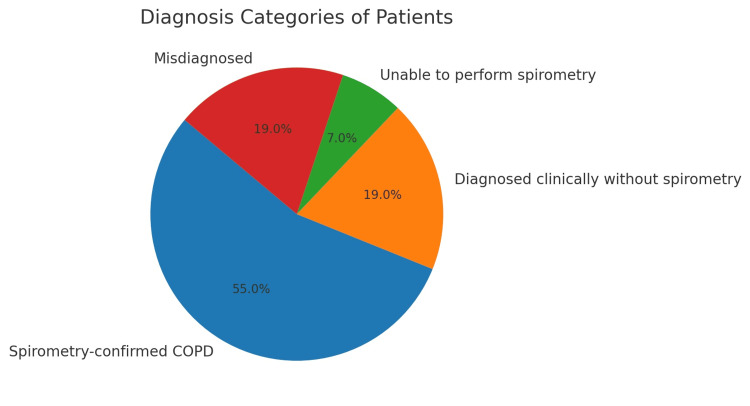
Diagnosis Categories of Patients COPD: Chronic obstructive pulmonary disease

A total of 1,452 electronic medical records were screened between January 2020 and May 2023 using the keyword “COPD.” Among these, 377 patients had a documented diagnosis of COPD. After excluding 221 patients due to incomplete data, 156 patients remained for detailed review. Of these, 86 patients (55%) had spirometry-confirmed COPD and were included in the final analysis. The remaining 70 patients were excluded for the following reasons: 30 (19%) were diagnosed clinically without spirometry, 11 (7%) were unable to perform spirometry, and 29 (19%) were misdiagnosed (three with asthma, eight with pre-COPD, and 18 with preserved ratio impaired spirometry [PRISm]). Patient selection and exclusion details are illustrated in Figure 1.

Demographic and clinical characteristics

The mean age of the study population at the time of analysis was 69.9 ± 8.6 years. The mean age at diagnosis was 63.6 ± 11.3 years, and the mean age at symptom onset was 63.2 ± 11.3 years, with an average delay of approximately four months between symptom onset and diagnosis. Of the 86 patients included in the study, 67 (77.9%) were male and 19 (22.1%) were female. This is also depicted in a tabular form as Table 1. Comorbidities were present in 71 patients (82.6%). Regarding smoking history, 36 patients (41.9%) were never-smokers. Among the remaining patients, 25 (29.1%) were classified as mild smokers (smoking index <100), five (5.8%) as moderate smokers (index 101-200), and 20 (23.3%) as heavy smokers (index >200).

**Table 1:** Patient Demographics and Characteristics

Description	Value
Number of participants	86
Females	19
Males	67
Mean age at the time of study (±SD)	69.94 (8.58)
Mean age at diagnosis (±SD)	63.7 (11.18)
Mean age at onset of symptoms (±SD)	63.25 (11.18)
Mean time taken for diagnosis	6 months

Distribution of etiotypes

Among male patients, the most prevalent etiotypes were COPD-C (tobacco or cannabis exposure) and COPD-P (pollution-related), followed by COPD-I (infection-related) and COPD-A (asthma overlap). None of the male patients were classified under developmental (COPD-D) or genetically determined (COPD-G) etiotypes. In contrast, all female patients (100%) were classified as having COPD-P. Smaller proportions of female patients also exhibited COPD-A (15.8%), COPD-I (5.3%), and COPD-D (15.8%). Similar to males, no female patients were classified under COPD-C or COPD-G. Exposure patterns differed markedly by sex. Male patients with COPD-P were more frequently exposed to outdoor air pollution (80.4%) than indoor sources (64.7%). Female patients were more commonly exposed to indoor air pollution (94.7%), particularly from biomass fuel, compared to outdoor exposure (57.9%). The full distribution of etiotypes by sex is summarized in Table 2.

**Table 2:** Distribution of Etiotypes Among Chronic Obstructive Pulmonary Disease (COPD) Patients by Sex

Etiotype	Male (n = 67)	Female (n = 19)
COPD-C (smoke)	50 (74.6%)	0 (0%)
COPD-P (pollution)	51 (76.1%)	19 (100%)
COPD-I (infection)	9 (13.4%)	1 (5.3%)
COPD-A (asthma overlap)	9 (13.4%)	3 (15.8%)
COPD-D (developmental)	0 (0%)	3 (15.8%)
COPD-G (genetic)	0 (0%)	0 (0%)

Single versus multiple etiotypes

Of the 86 patients included in the study, 33 (38.4%) were classified as having a single etiotype, while 53 patients (61.6%) exhibited two or more etiotypes. A statistically significant association was observed between the presence of multiple etiotypes and greater spirometric severity (p = 0.046). However, no significant associations were found between the number of etiotypes and either the degree of breathlessness (p = 0.8223) or frequency of exacerbations (p = 0.6269). Detailed associations between single versus multiple etiotypes and clinical severity indicators are provided in Table 3.

**Table 3:** Association of Etiotype Number (Single vs Multiple) With Clinical Severity mMRC: modified Medical Research Council

Outcome	Single etiotype (n = 33)	Multiple etiotypes (n = 53)	p-value	Chi-square values
Mild–Moderate obstruction	21	22	0.046	3.983
Severe–Very severe obstruction	12	31		
Mild–Moderate breathlessness (mMRC 1–2)	25	39	0.8223	0.05
Severe–Very severe breathlessness (mMRC 3–4)	8	14		
Non-frequent exacerbators	24	41	0.6269	0.236
Frequent exacerbators	9	12		

Smoking status and clinical outcomes

No statistically significant association was observed between smoking status and spirometric severity (p = 0.08), degree of breathlessness (p = 0.649), or frequency of exacerbations (p = 0.687). These findings suggest that smoking history, while relevant, may not be the sole determinant of disease severity in this population. A detailed breakdown of clinical outcomes based on smoking status is presented in Table 4.

**Table 4:** Association of Smoking Status With Clinical Severity

Outcome	Smokers (n = 50)	Never-smokers (n = 36)	p-value	Chi-square values
Mild–Moderate obstruction	21	22	0.08	3.058
Severe–Very severe obstruction	29	14		
Mild–Moderate breathlessness	36	13	0.649	0.207
Severe–Very severe breathlessness	14	9		
Non-frequent exacerbators	37	28	0.687	0.1618
Frequent exacerbators	13	8		

Pollution exposure and clinical outcomes

Among the 86 patients included in the analysis, 70 (81.4%) had exposure to indoor or outdoor pollution. No statistically significant associations were found between pollution exposure and spirometric severity (p = 0.65), degree of breathlessness (p = 0.565), or frequency of exacerbations (p = 0.225). These results suggest that while pollution is a major contributor to COPD etiology, it may not directly correlate with clinical severity indicators in isolation. Detailed outcome data related to pollution exposure are presented in Table 5.

**Table 5:** Association of Pollution Exposure With Clinical Severity

Outcome	Pollution Exposure (n = 70)	No Pollution Exposure (n = 16)	p-value	Chi-Square values
Mild–Moderate obstruction	35	9	0.65	0.204
Severe–Very severe obstruction	35	7		
Mild–Moderate breathlessness	53	11	0.565	0.332
Severe–Very severe breathlessness	17	5		
Non-frequent exacerbators	54	10	0.225	1.467
Frequent exacerbators	16	6		

## Discussion

COPD remains a leading cause of morbidity and mortality globally [6,7]. Accurate diagnosis requires not only persistent respiratory symptoms, such as cough, dyspnea, wheeze, and sputum production, but also spirometric confirmation of post-bronchodilator airflow obstruction. This is crucial to distinguish COPD from other conditions like asthma and bronchiectasis.

In our study, only 86 of 156 patients (55.1%) clinically diagnosed with COPD met spirometric criteria. Notably, 18.6% were misdiagnosed despite compatible symptoms and risk exposures, suggesting conditions such as pre-COPD, PRISm, asthma, or bronchiectasis. This underscores the importance of objective confirmation via spirometry. Treating patients with pre-COPD or PRISm using bronchodilators remains unsupported by current evidence [7-9], and overuse of inhaled corticosteroids (ICS) in non-asthmatic COPD may pose unnecessary risks. Additionally, 19% of patients had not undergone spirometry at diagnosis, reflecting a potential gap in clinical practice. Another 7% were unable to perform spirometry, pointing to the need for validated alternative diagnostic tools in such cases. The average age of COPD diagnosis in our cohort (63.6 years) was higher than the global average (~53 years) [10], possibly reflecting delayed recognition or limited access to healthcare. COPD prevalence was higher among males (77.9%), consistent with previous Indian studies [11]. Notably, none of the female patients reported a history of smoking, highlighting the substantial contribution of non-smoking-related risk factors to COPD in the Indian population [12].

This study is among the first to apply the GOLD 2023 etiotype classification in an Indian setting. Most male patients exhibited overlapping etiologies - primarily COPD-C (smoking-related) and COPD-P (pollution-related). All female patients were categorized under COPD-P, reflecting high exposure to indoor biomass smoke from traditional cooking stoves (chulhas), in line with earlier reports [13]. The uniform classification of female patients under COPD-P likely reflects true exposure differences, as detailed histories of indoor air exposures were collected, including regular incense use and type of cooking fuel. We also identified incense smoke as a possible contributor, particularly among male patients. While its role in COPD remains debated, emerging evidence suggests incense combustion releases particulate matter and volatile compounds that may trigger oxidative stress and airway inflammation [14-16]. Studies among temple workers have shown increased respiratory symptoms with prolonged exposure [17], though others report no significant association [18]. Animal studies further support the potential for incense-related lung damage [19-21]. However, larger human studies are needed for definitive conclusions. The average age of COPD diagnosis in our cohort was higher than global averages, likely reflecting delayed recognition of the disease in Indian settings. Contributing factors may include limited availability and accessibility of spirometry, under-recognition of non-smoking exposures such as biomass smoke, and low clinical awareness of COPD in populations exposed to indoor and outdoor pollutants.

A key finding of this study was the significant association between multiple etiotypes and more severe spirometric obstruction (p = 0.046), suggesting that cumulative exposures may accelerate disease progression. Biologically, repeated exposures to tobacco smoke, biomass fuel, outdoor pollution, and recurrent infections can lead to additive oxidative stress, chronic airway inflammation, epithelial injury, mucus hypersecretion, and small airway remodeling, providing a plausible mechanism for the observed relationship between multiple etiotypes and greater airflow limitation [1-3,20]. This relationship has not been widely reported and may have implications for risk stratification and preventive strategies. Contrary to previous research suggesting a more favorable disease course in non-smokers [22], our study did not find significant differences in lung function, symptom severity, or exacerbation frequency between smokers and never-smokers. This may reflect the dominant role of environmental and infectious exposures in our cohort. The absence of significant associations between certain exposures and clinical severity may reflect the limited sample size of our cohort or potential misclassification of self-reported exposure data. However, consistent with existing literature, we observed a significant association between lower FEV₁ and more frequent exacerbations (p = 0.035) [23].

Limitations

This study has several limitations. It was observational and based on retrospective medical record review and patient interviews, which may introduce selection and recall bias. Exposure data were self-reported and not corroborated by objective environmental measurements. The small female subgroup (n = 19) limits the generalizability of sex-specific findings, and results related to female patients should therefore be interpreted with caution. In addition, the classification of pollution exposure relied on patients’ perceptions rather than standardized indices or quantitative assessments. As a cross-sectional study, the associations observed are observational and hypothesis-generating and should not be interpreted as causal. Future research incorporating objective exposure metrics (e.g., household or ambient PM2.5 levels) and multicentric prospective designs would help validate the etiotype framework and assess its prognostic relevance for outcomes such as FEV₁ decline, exacerbation frequency, and mortality.

## Conclusions

COPD in the Indian population exhibits significant etiological diversity, with a notable prevalence of non-smoking-related cases, particularly among women. Biomass exposure and indoor air pollutants, including traditional cooking stoves and incense smoke, emerged as key contributors. Most patients exhibited overlapping etiotypes as per the GOLD 2023 classification, with COPD-C and COPD-P being most common. Importantly, the presence of multiple etiotypes was significantly associated with greater spirometric severity, underscoring the cumulative impact of varied exposures on lung function. However, no significant associations were found between etiotype multiplicity and either symptom burden or exacerbation frequency.

These findings highlight the urgent need for routine spirometry for accurate diagnosis, greater clinical awareness of non-traditional COPD risk factors and population-specific phenotyping to guide personalized care. Future research should explore the impact of individual and combined exposures using objective pollution metrics and long-term follow-up.
